# Factors Influencing Pediatric Residency Graduates' Decisions to Take the Initial Board Examination and Their Likelihood of Success

**DOI:** 10.7759/cureus.85784

**Published:** 2025-06-11

**Authors:** Joann Carlson, Natalie Torres, Anna Petrova

**Affiliations:** 1 Pediatrics, Rutgers Robert Wood Johnson Medical School, New Brunswick, USA

**Keywords:** american board of pediatrics certifying examination (abp-ce), factors predicting taking and passing the abp-ce, pediatric residents, rate of passing initial abp-ce, rate of taking initial abp-ce

## Abstract

Introduction

The American Board of Pediatrics Certifying Examination (ABP-CE) is not just a component, but the cornerstone of general pediatricians' professional training. Passing the ABP-CE on the first attempt is essential for maintaining the quality of residency programs and ensuring the successful practice of pediatric graduates.

Objectives

This study is the first to investigate how various individual factors and the residency program environment, particularly during the unprecedented challenges posed by the COVID-19 pandemic, impact the performance of pediatric residency graduates on the initial board examination, specifically regarding not taking or not passing the ABP-CE.

Methods

We conducted an analysis of graduates from a single pediatric residency program between 2014 and 2024. The analysis included various factors such as the graduates' age, sex, type of degree, medical school attended, fellowship acceptance, scores from the United States Medical Licensing Examination (USMLE) Step 1 and in-training examinations (ITEs), and the impact of training during the COVID-19 pandemic. We employed descriptive statistics and stepwise logistic regression models for our analysis. The findings are presented in terms of percentages, means, and odds ratios (ORs) with a 95% confidence interval (95% CI). A p-value of less than 0.05 was considered statistically significant.

Results

Among the 121 program graduates, 98 (80.9%) attempted the initial ABP-CE, with a pass rate of 85.7%. None of the demographic or educational characteristics of the residents were identified as predictors for not taking or not passing the initial ABP-CE. A lower score on the third-year ITE was associated with not taking the examination, with an OR of 0.96 (95% CI: 0.93, 0.99; P < 0.03), and not passing the examination, with an OR of 0.92 (95% CI: 0.88, 0.97; P < 0.01). Moreover, residents who did not pass the ABP-CE were more likely to have been exposed to COVID-19 during training than those who successfully passed the examination (OR: 4.06; 95% CI: 1.44, 11.5; P < 0.01). Training during COVID-19 did not significantly impact the decision of graduates to take the initial ABP-CE.

Conclusion

Our study offers valuable insights for pediatricians in training, educators, and researchers about the factors predicting outcomes on the initial ABP-CE for pediatric program graduates. It reveals that training during the COVID-19 pandemic had no effect on the decision of residents to skip taking the initial ABP-CE but increased the chance for ABP-CE failure by nearly four times compared to program graduates who successfully passed the initial ABP-CE. Furthermore, the study indicated that each point reduction in scores on the third-year ITE corresponded to an average increase of 4% in the likelihood of not taking the ABP-CE and an 8% increase in the likelihood of not passing it.

## Introduction

The American Board of Pediatrics Certifying Examination (ABP-CE) is often used to evaluate the professional competency and career prospects of graduates from pediatric residency programs [1]. Additionally, the Accreditation Council for Graduate Medical Education (ACGME) relies on the first-time passing rate of the ABP-CE to monitor the quality of residency programs and to maintain their accreditation [2]. In general, obtaining board certification in any specialty is required to practice at hospitals and to be credentialed by insurance companies [3].

The pass rate for the ABP-CE is notably influenced by the structure of residency programs and the unique characteristics of pediatric residents [4]. Moreover, taking the initial board examination could also reflect the self-confidence of residents regarding their readiness for practicing medicine. Analysis of publicly available databases has shown that passing the certifying examination is associated with certain educational programming, such as participating in weekly lectures or conferences, having a strong presence of full-time faculty, and a significant number of graduates coming from US medical schools [5]. Some studies have investigated the size and location of pediatric residency programs to determine their impact on the ABP-CE passing rates. The findings are mixed, with some studies reporting no effect [5], while others highlight a positive impact associated with larger programs [6]. Recent data indicate that pediatric residents who graduate from programs located in major urban areas or regions with multiple pediatric residency programs tend to achieve higher scores on certifying examinations [7]. Research examining the individual resident-related factors that predict success on the first attempt of the ABP-CE remains limited, particularly in relation to performance on the United States Medical Licensing Examination (USMLE) Steps and the annual in-service training examination (ITE) [8-10]. A direct correlation has been established between the passing rates of the ABP-CE and the scores of the USMLE Step 1 [8,9] and Step 2 [8] examinations. However, the impact of the USMLE Step 2 and annual ITE scores appears to be insignificant [8-10]. Additionally, the predictive power of first-year ITE scores on ABP-CE passing rates is minimal when compared to that of third-year ITE scores [10].

The recent ACGME report displaying the negative trend toward reduction of the first ABP-CE passing rate from 91% in 2018 to 87% in 2019-2020 and 81%-80% in 2021-2022 [11,12] indicates the need for developing and implementing a prompt system to safeguard the success of the initial certifying examination by the US pediatric residency program graduates. The changes in the education environment during the COVID-19 pandemic may have contributed to the decline in the ABP-CE passing rate.

Research in the pediatric postgraduate education field features the importance of identifying significant predictors of pediatric residents' decisions to take and pass the first-time ABP-CE. However, research that explores various factors predicting first-time success on the ABP-CE is limited. To our knowledge, no study has analyzed factors predicting the graduate's decision to take the ABP-CE or other boards for medical specialties.

The current study was designed to identify the factors that significantly predict whether pediatric program graduates pass or choose not to take the initial ABP-CE. This is the first study to analyze demographic characteristics, educational background, and the scores from USMLE Step 1 and ITEs conducted annually, as well as the impact of training during the COVID-19 pandemic on first-time ABP-CE outcomes. The findings of this study will hopefully enhance our understanding of the factors that influence passing the ABP-CE and, for the first time, will identify those that may affect a program graduate's decision not to take the ABP-CE immediately after graduation. This knowledge could be crucial in developing educational strategies to better prepare future graduates for the ABP-CE throughout their three years of training in pediatric residency programs.

## Materials and methods

Design

We conducted a retrospective population-based cohort study of all pediatric residents who had already graduated from a single university-affiliated pediatric residency program between 2014 and 2024. Numerical residency program files were utilized to create a de-identified database for the required analysis.

Participants and setting

The program features 33 residents, with 11 residents per year, which meets the definition of a medium-sized pediatric residency program [13]. It boasts more than 140 full-time faculty members who cover 20 pediatric subspecialties, providing residents with exposure to a diverse pediatric population facing various medical conditions of differing severity. The program is situated in a city with a population of nearly 60,000, of which 27.3% are children under the age of 18 [14].

Inclusion and exclusion criteria

We included residents who had already graduated from the program between 2014 and 2024. Residents who did not complete the program were excluded from the study. A total of 11 residents graduated in most years, with the exceptions of 2017, which had 12 graduates, and 2020, which had 10 graduates.

Data collection tool

The information collected provided a clear distinction of the ABP-CE status for program graduates, specifically identifying whether they participated in the examination and, for those who did, whether they passed or failed. We also collected demographic information from resident records, including their sex and age, as well as their type of medical degree, which could be either a Medical Doctor (MD) or a Doctor of Osteopathic Medicine (DO). Additionally, we gathered data on the medical school from which they graduated, distinguishing between American Medical Graduates (AMGs) and International Medical Graduates (IMGs). We also noted whether they were accepted into a fellowship program and recorded their absolute scores on the USMLE Step 1 examination and the annual in-training examinations (ITEs) conducted during their first, second, and third years. The WHO-declared time of initiation of the COVID-19 pandemic [15] was used to define years of training during the COVID-19 pandemic.

Data analysis

Data was analyzed based on the graduates' first-time ABP-CE status: whether they took or did not take the ABP-CE and whether they passed or failed. The collected factors were compared between the study groups using the Chi-square test for categorical variables and analysis of variance (ANOVA) for continuous variables. We utilized a t-test for dependent samples to assess the significance of changes in the ITE scores over the three years of the residency program. Furthermore, we used correlation to present the association between the ITE annual scores. Finally, we constructed stepwise logistic regression models to identify significant predictors for not passing the ABP-CE (Model 1) and not taking the ABP-CE (Model 2). According to the rules-of-thumb (10 subjects for every independent variable in the model), the number of 121 graduates in this study will be sufficient for inclusion in the model up to 12 pre-selected variables.

The results of our statistical analysis were presented as proportions (%), means, mean differences, and correlation (r) coefficient. The exponential function was utilized to transform regression coefficients (β) into the odds ratios (ORs). Each statistical data point was accompanied by a robust 95% confidence interval (95% CI). A p-value of less than 0.05 was deemed statistically significant. Our analysis was conducted using STATISTICA version 14.1 (TIBCO Software, Inc., Palo Alto, CA).

Ethical consideration

The study was approved by Rutgers Robert Wood Johnson Medical School Institutional Review Board (approval number: PRO2023001166). No consent was required for retrospective analysis of de-identified subjects who had already graduated residency program.

## Results

Between 2014 and 2024, 121 graduates of the pediatric residency program were included in this study. Out of these graduates, 98 (80.9%) took the initial ABP-CE, and among them, 84 (85.7%) successfully passed the examination.

Table 1 presents a detailed summary of the distribution and quantities of the variables examined in this study. All continuous variables, which include age and scores from the annual ITEs, as well as the USMLE Step 1, exhibited a normal distribution. Most graduates from the pediatric program were women, with an average age of nearly 30 years and an MD degree. Approximately one-third of these graduates were accepted into various fellowship programs. It is noteworthy that among the 121 graduates, 54 (44.6%) completed their residency during the COVID-19 pandemic, including 21 (38.9%) who were exposed to COVID-19 for a duration of one year, 21 (38.9%) for two years, and 12 (22.2%) for three years.

**Table 1:** Description of collected factors in studied residents CI: confidence interval, COVID-19: coronavirus disease 2019, IMG: International Medical Graduate, ITE: in-training examination, USMLE: United States Medical Licensing Examination

Factors	Number	Mean (95% CI)
Age, years	121	30.5 (30.5, 31.3)
Female sex, %	121	78.5 (70.1, 85.5)
Medical doctor, %	121	66.9 (57.8, 74.2)
Fellowship acceptance, %	121	35.5 (27.1, 44.8)
IMG, %	121	5.8 (2.4, 11.7)
Training during the COVID-19 pandemic, %	121	44.6 (35.6, 53.9)
ITE score, 3rd year	117	156.9 (153.8, 160.0)
ITE score, 2nd year	118	149.0 (143.3, 154.7)
ITE score, 1st year	119	112.0 (103.5, 120.5)
USMLE Step 1 score	109	217.5 (214.6 220.5)

**Figure 1:**
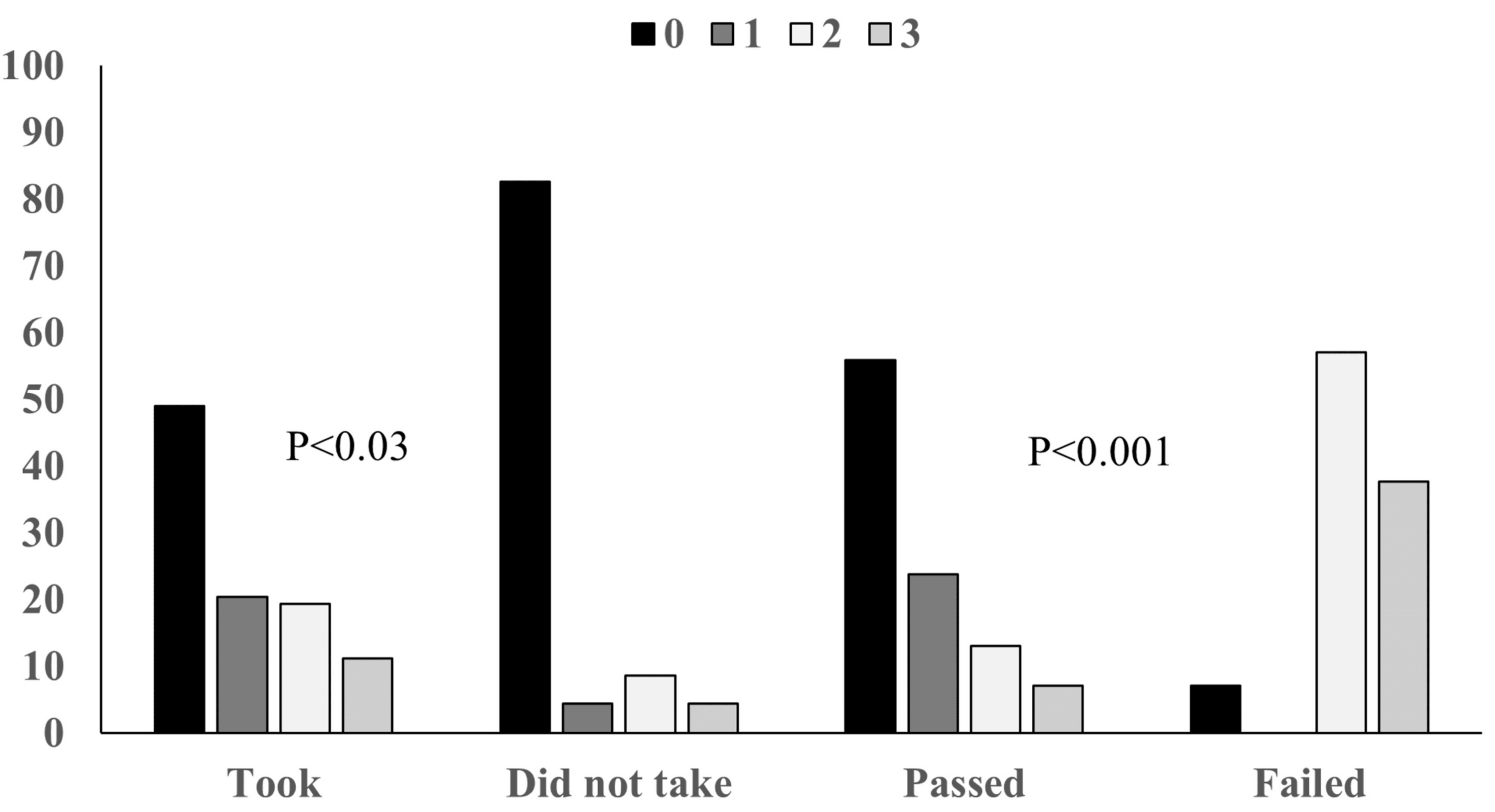
Comparison of years of residency (0, 1, 2, and 3) during the COVID-19 pandemic based on ABP-CE outcomes ABP-CE: American Board of Pediatrics Certifying Examination, COVID-19: coronavirus disease 2019

Figure 1 shows the years of residency during the COVID-19 pandemic and their impact on ABP-CE outcomes. It compares program graduates who took the ABP-CE with those who did not, and it looks at participants who passed the ABP-CE compared to those who did not. The data reveals that more graduates who attempted the ABP-CE faced challenges due to the COVID-19 pandemic than those who chose not to take the examination. Additionally, graduates who did not pass the ABP-CE were more likely to have completed their two to three years of residency during the pandemic than those who passed.

Relationship between ITE scores obtained within the first, second, and third years

The paired t-test revealed a significant increase in the ITE scores for residents from their first to their second year, with a mean difference of 43.0 (95% CI: 30.8, 53.2; P < 0.0001). The ITE increase from the second to the third year was 7.1 (95% CI: 0.74, 13.4; P < 0.03). There was no correlation between ITE scores in the first year and scores in the second year (r = 0.18, P = 0.36) or third year (r = 0.17, P = 0.42). However, there was a significant correlation between scores in the second and third years (r = 0.43, P < 0.001).

Comparison of pediatric graduates' characteristics based on the ABP-CE status

Table 2 compares the characteristics of graduates who passed the ABP-CE with those who failed, as well as between graduates who took the initial ABP-CE and those who did not. The analysis revealed differences in third-year ITE scores and training received during the COVID-19 pandemic between the graduates who passed and those who failed the ABP-CE. Furthermore, there were significant differences in first-year and third-year ITE scores and training during the COVID-19 pandemic between graduates who participated in the initial ABP-CE and those who did not.

**Table 2:** Group-based comparison of studied factors (mean or % with 95% CI) *: <0.05-0.001, ±: <0.02-0.01 ABP-CE: American Board of Pediatrics Certifying Examination, CI: confidence interval, COVID-19: coronavirus disease 2019, IMG: International Medical Graduate, ITE: in-training examination, MD: Medical Doctor, USMLE: United States Medical Licensing Examination

Factors	Study groups based on the ABP-CE status			
	Model 1		Model 2	
	Passed	Failed	Took	Did not take
	N = 84	N = 14	N = 98	N = 23
Age, years	31 (30, 32)	31 (30, 32)	32 (31, 33)	31 (30, 31)
Female, %	81.0 (70.9, 88.7)	78.6 (49.2, 95.3)	80.6 (53.1, 74.5)	69.6 (47.1,86.8)
MD, %	64.3 (53.1, 74.5)	57.1 (28.9, 82.1)	64.3 (54.0, 73.1)	78.3 (56.3, 92.5)
IMG, %	6.0% (2.0, 13.4)	7.1 (0.20, 33.9)	6.1 (2.3, 12.9)	4.4 (0.11, 22.0)
Fellowship, %	36.9 (26.6, 48.1)	28.6 (8.4, 58.1)	35.7 (26.3, 46.0)	34.8 (16.4, 57.3)
ITE score, 1st year	116 (106, 126)	124 (116, 132)	117 (109, 126)	90 (64, 115) ±
ITE score, 2nd year	152 (145, 156)	138 (132, 143)	150 (144, 156)	146 (130, 162)
ITE score, 3rd year	161 (134, 153)	144 (134, 153)*	159 (155, 162)	149 (144, 155) ±
USMLE score	219 (215, 222)	212 (205, 220)	218 (215, 221)	216 (208, 224)
COVID-19, %	44.1 (33.2, 55.3)	92.9 (66.2-99.8)*	51.0 (40.7, 61.3)	17.4 (5.0, 39.8) ±

Factors that showed significant difference were selected for inclusion in the regression models that were designed to identify their independence in predicting ABP-CE falling (Model 1) and ABP-CE not taking (Model 2). Model 1 showed an association between not passing ABP-CE and a decrease in third-year ITE scores (OR: 0.92; 95% CI: 0.88, 0.97; P < 0.01). Additionally, exposure to the COVID-19 environment during residency training was associated with higher odds of not taking the initial ABP-CE (OR: 4.06; 95% CI: 1.44, 11.5; P < 0.01). Model 2 identified that a decrease in third-year ITE scores predicted not taking the initial ABP-CE (OR: 0.96; 95% CI: 0.93, 0.99; P < 0.03), whereas first-year ITE scores showed no significant prediction (OR: 0.99; 95% CI: 0.98, 1.01; P = 0.57). Furthermore, residency program graduates who did not take the initial ABP-CE were less likely to have been trained during the COVID-19 pandemic compared to those who did take the examination, with an OR of 0.46 (95% CI: 0.25, 0.87; P < 0.01).

## Discussion

In this study, we examined factors predicting either failing or not taking the initial ABP-CE by university-based pediatric residency program graduates from 2014 to 2024. Our study found that the risk of failing the initial ABP-CE increased nearly fourfold among program graduates who were exposed to the COVID-19 pandemic during their residency training, likely affecting them significantly for two to three years. COVID-19-related changes in the educational environment [16], including switching to virtual lectures and decreasing clinical volume of in-person pediatric care [17], could be responsible for the increased risk for ABP-CE falling as defined in our study. Furthermore, the COVID-19 pandemic negatively impacted the passing of pediatric gastroenterology and pulmonology board certification examinations [18]. However, exposure to COVID-19 did not affect the passing rate of the emergency medicine fellows' board examinations [18]. It is important to know that the COVID-19 pandemic has the potential to profoundly influence the psychological well-being of graduates. Pediatricians preparing for their initial certifying examinations have shared notable challenges, revealing how the pandemic has negatively impacted their career choices and job search experiences [19].

Our analysis indicates that while graduates' demographics and their scores on the first-year and second-year ITEs do not significantly influence performance on the ABP-CE, the third-year ITE scores emerge as a critical factor. Specifically, even a slight decrease of one point in the third-year ITE score can lead to an approximately 8% increase in the risk of not passing the initial ABP-CE. This finding aligns with earlier research from 2001 to 2005, which highlighted the vital role that third-year ITE scores play in predicting successful outcomes in the pediatric board examination [10]. Additionally, a study involving 17 graduates from large pediatric residency programs in 2003 established a direct correlation between ABP-CE scores and both the first-year and third-year ITE scores [20]. However, a study of 170 pediatric program graduates from 2005 to 2008 determined a strong correlation between all three years of ITE and board certification scores [21].

Our study did not support previous reports [8,9] that indicated USMLE Step 1 scores in the prediction of performance on the ABP-CE. With the shift to a pass/fail scoring system, the significance of USMLE Step 1 scores in resident selection and, therefore, their ability to predict ABP-CE performance has decreased. Furthermore, the American Board of Internal Medicine (ABIM) indicated better prediction of ABIM certifying exam results by the third-year ITE rather than by USMLE Step scores [22,23] or demographic factors [23,24].

Delaying the ABP-CE can lead to a lower passing rate [25], highlighting the importance of understanding the factors influencing pediatric graduates' decisions not to take the examination on their first attempt. To the best of our knowledge, this study is the first to analyze factors predicting the failure to take examinations by graduates of pediatric or other residency programs. Our study recorded that 19% of graduates opted out of taking the ABP-CE after completing their program. We found an association where a decrease of one point in the scores of the third-year ITE resulted in a nearly 4% increase in the likelihood of graduates not taking the initial ABP-CE. The graduates who did take the initial ABP-CE were more likely to be exposed to COVID-19 during their residency compared to those who did not take the examinations. This difference may be due to the inclusion of residents who failed the examination in the comparison group, as their residency coincided with the COVID-19 pandemic. To address this, we conducted a regression analysis excluding graduates who failed the ABP-CE from the group of those who took the initial board examinations. The results confirmed a reduction in third-year ITE scores (OR: 0.95; 95% CI: 0.94, 0.99; P < 0.01). However, there was no significant difference in COVID-19 exposure between the graduates who took the examination and those who did not (OR: 1.76; 95% CI: 0.31, 1.03; P = 0.07).

This study has potential limitations. First, there was a limited number of participants representing the study outcomes, such as ABP-CE failing and not taking. A larger population of pediatric residency program graduates or from multiple programs would be better to ensure statistical precision, although our study's overall number of program graduates fulfilled the regression model-building requirements [26]. Second, it is evident that our study findings depended on the types of predictors. Annual changes in the clinical environment and program curriculum between 2014 and 2024, which we did not analyze in this study, except for the training time during the COVID-19 pandemic, could confound the role of the included factors in predicting the study outcomes. Third, the external validity of the study's findings that are based on data from a medium-sized program could be a limitation for other-sized pediatric residency programs.

## Conclusions

Board certification plays an important role in measuring the professional competence of graduates and the quality of residency programs. Since not all graduates take the ABP-CE immediately after graduation, it becomes essential to explore the factors that influence pediatric graduates' decisions on this matter. Not one of the individual resident-related factors predicted the graduate's decision not to take the initial ABP-CE or fail the ABP-CE among those who took the board examination. Our study findings demonstrated the notable effect of training during the COVID-19 pandemic on predicting the likelihood of failing the initial board certifying examination. Additionally, the study found that a reduction in scores on the third-year in-training examination predicted both not taking and failing the ABP-CE.

Enhancing the ability of third-year residents to substantially improve ITE scores could be essential for the initial take and pass of the board examination. The negative effect of the COVID-19 pandemic on the success on initial ABP-CE could be due to changes in the educational curriculum, reduction in exposure of pediatric residents to primary care, and hospital admissions. This study highlights the need for the development of rescue strategies specifically for future events to preserve the educational structure of pediatricians in training.

## References

[1] Frank JR, Snell LS, Cate OT (2010). Competency-based medical education: theory to practice. Med Teach.

[2] (2023). ACGME Program Requirements for Graduate Medical Education in Pediatrics. https://www.acgme.org/globalassets/pfassets/programrequirements/2025-prs/320_pediatrics_2025.pdf..

[3] Greenky D, Reddy P, George P (2021). Rethinking the initial board certification exam. Med Sci Educ.

[4] The American Board of Pediatrics. (2024). Exam pass rates. https://www.abp.org/content/exam-pass-rates.

[5] Atsawarungruangkit A (2015). Residency program characteristics that are associated with pass rate of the American Board of Pediatrics certifying exam. Adv Med Educ Pract.

[6] Falcone JL (2014). Size matters: the importance of residency program size to pass rates on the American Board of Pediatrics Certifying Examination. Clin Pediatr (Phila).

[7] Falcone JL (2014). City population size is associated with examinee outcomes on the American Board of Pediatrics Certifying Examination. Clin Pediatr (Phila).

[8] Welch TR, Olson BG, Nelsen E, Beck Dallaghan GL, Kennedy GA, Botash A (2017). United States Medical Licensing Examination and American Board of Pediatrics certification examination results: does the residency program contribute to trainee achievement. J Pediatr.

[9] McCaskill QE, Kirk JJ, Barata DM, Wludyka PS, Zenni EA, Chiu TT (2007). USMLE Step 1 scores as a significant predictor of future board passage in pediatrics. Ambul Pediatr.

[10] Althouse LA, McGuinness GA (2008). The in-training examination: an analysis of its predictive value on performance on the general pediatrics certification examination. J Pediatr.

[11] (2024). The complete guide to the pediatric board exam. https://explore.medstudy.com/blog/pediatric-board-exam-survival-guide.

[12] (2023). Initial certifying examination. https://www.abp.org/sites/abp/files/pdf/exam-pass-rates-init-cert.pdfm.

[13] Jennings RM, Thompson LA, Townsend CL, Stashwick CA, Goodman DC (2003). The relationship between pediatric residency program size and inpatient illness severity and diversity. Arch Pediatr Adolesc Med.

[14] (2025). QuickFacts: New Brunswick city, New Jersey. https://www.census.gov/quickfacts/fact/table/newbrunswickcitynewjersey/PST045222.

[15] Cucinotta D, Vanelli M (2020). WHO declares COVID-19 a pandemic. Acta Biomed.

[16] (2023). Should pediatricians fret their falling board scores?. https://www.medscape.com/viewarticle/987220?form=fpf.

[17] Naifeh MM, Stevenson MD, Abramson EL, Aston CE, Li ST (2021). Early impact of the COVID-19 pandemic on pediatric resident workforce. Pediatrics.

[18] Ngo TL, Yanek L, Caglar D, Bailey J, Roskind CG, Langhan M (2024). Medical knowledge acquisition during a pandemic: pediatric subspecialty in-training examination and board certification exam passing rate. Acad Pediatr.

[19] Li ST, Turner AL, Naifeh MM (2023). COVID-19 pandemic impact on pediatricians entering the pediatric workforce. Acad Pediatr.

[20] Chase LH, Highbaugh-Battle AP, Buchter S (2012). Residency factors that influence pediatric in-training examination score improvement. Hosp Pediatr.

[21] Aeder L, Fogel J, Schaeffer H (2010). Pediatric board review course for residents "at risk". Clin Pediatr (Phila).

[22] Kay C, Jackson JL, Frank M (2015). The relationship between internal medicine residency graduate performance on the ABIM certifying examination, yearly in-service training examinations, and the USMLE Step 1 examination. Acad Med.

[23] Rayamajhi S, Dhakal P, Wang L, Rai MP, Shrotriya S (2020). Do USMLE Steps, and ITE score predict the American Board of Internal Medicine certifying exam results?. BMC Med Educ.

[24] Seaberg PH, Kling JM, Klanderman MC (2023). Resident factors associated with American Board of Internal Medicine certification exam failure. Med Educ Online.

[25] (2024). Licensing and board certification: what residents need to know. January.

[26] Wilson CR, Voorhis V, Morgan BL (2007). Understanding power and rules of thumb for determining sample sizes. Tutor Quant Methods Psychol.

